# The Practitioner’s Pharmacopœia and Universal Formulary: Containing 2000 Classical Prescriptions Selected from the Practice of the Most Eminent British and Foreign Medical Authorities

**Published:** 1856-03

**Authors:** 


ART. VII.—*The Practitioner's Pharmacopœia and Universal Formulary: containing* 2000 *Classical Prescriptions selected from the practice of the most eminent British and Foreign Medical Authorities.* With an abstract of the three British Pharmacopœias, and much other useful information for the Practitioner and Student. By John Foote, M. R. C. S., London. With corrections and additions by an American Physician. New York: S. S. & Wm. Wood. 1855.

Some one has laid felonious hands on our Beasley's Prescription Book, upon which we placed some value. This is but a poor substitute for it. It is a *physic-book,* informing us what "is good" for what, got up on something the plan of a cookery book. We received our copy from the publishers, through A. I. Mathews & Co., Druggists. If any reader wishes a copy he can order it through that firm.

